# Association between antenatal corticosteroid treatment and severe adverse events in pregnant women

**DOI:** 10.1186/s12916-023-03125-w

**Published:** 2023-10-31

**Authors:** Hui-Ju Tsai, Beth I. Wallace, Akbar K. Waljee, Xiumei Hong, Sheng-Mao Chang, Yi-Fen Tsai, Mei-Leng Cheong, Ann Chen Wu, Tsung-Chieh Yao

**Affiliations:** 1 Institute of Population Health Sciences, National Health Research Institutes, Zhunan, Taiwan; 2 National Tsing-Hua University, Hsinchu, Taiwan; 3 Center for Clinical Management Research, Lieutenant Colonel Charles S. Kettles VA Medical Center, Ann Arbor, MI USA; 4 University of Michigan, Ann Arbor, MI USA; 5 Institute for Healthcare Policy and Innovation, Ann Arbor, MI USA; 6 Department of Population, Family and Reproductive Health, Center On Early Life Origins of Disease, Johns Hopkins University Bloomberg School of Public Health, Baltimore, MD USA; 7 Department of Statistics, National Taipei University, Taipei, Taiwan; 8 Department of Obstetrics and Gynecology, Cathay General Hospital, Taipei, Taiwan; 9 Harvard Medical School, Boston, MA USA; 10 Division of Allergy, Asthma, and Rheumatology, Department of Pediatrics, Chang Gung Memorial Hospital, 5 Fu-Hsin Street, Kweishan, Taoyuan, 33305 Taiwan; 11 School of Medicine, Chang Gung University College of Medicine, Taoyuan, Taiwan

**Keywords:** Antenatal, Corticosteroids, Sepsis, Heart failure, Gastrointestinal bleeding

## Abstract

**Background:**

Antenatal corticosteroids are considered the standard of care for pregnant women at risk for preterm birth, but studies examining their potential risks are scarce. We aimed to estimate the associations of antenatal corticosteroids with three severe adverse events: sepsis, heart failure, and gastrointestinal bleeding, in pregnant women.

**Methods:**

Of 2,157,321 pregnant women, 52,119 at 24 weeks 0/7 days to 36 weeks 6/7 days of gestation were included in this self-controlled case series study during the study period of 2009–2018. We estimated incidence rates of three severe adverse events: sepsis, heart failure, and gastrointestinal bleeding. Conditional Poisson regression was used to calculate incidence rate ratios (IRRs) for comparing incidence rates of the adverse events in each post-treatment period compared to those during the baseline period among pregnant women exposed to a single course of antenatal corticosteroid treatment.

**Results:**

Among 52,119 eligible participants who received antenatal corticosteroid treatment, the estimated incidence rates per 1000 person-years were 0.76 (95% confidence interval (CI): 0.69–0.83) for sepsis, 0.31 (95% CI: 0.27–0.36) for heart failure, and 11.57 (95% CI: 11.27–11.87) for gastrointestinal bleeding. The IRRs at 5 ~ 60 days after administration of antenatal corticosteroids were 5.91 (95% CI: 3.10–11.30) for sepsis and 4.45 (95% CI: 2.63–7.55) for heart failure, and 1.26 (95% CI: 1.02–1.55) for gastrointestinal bleeding; and the IRRs for days 61 ~ 180 were 2.00 (95% CI: 1.01–3.96) for sepsis, 3.65 (95% CI: 2.14–6.22) for heart failure, and 1.81 (95% CI: 1.56–2.10) for gastrointestinal bleeding.

**Conclusions:**

This nationwide population-based study suggests that a single course of antenatal corticosteroids is significantly associated with a 1.3- to 5.9-fold increased risk of sepsis, heart failure, and gastrointestinal bleeding in pregnant women. Maternal health considerations, including recommendations for adverse event monitoring, should be included in future guidelines for antenatal corticosteroid treatment.

**Supplementary Information:**

The online version contains supplementary material available at 10.1186/s12916-023-03125-w.

## Background

Antenatal corticosteroid treatment in pregnant women has been used for accelerating fetal lung maturity and reducing the incidence of neonatal respiratory distress syndrome since 1972 [1, 2]. This practice has been considered the standard treatment and has been increasingly used for decades, particularly after the conclusions made by the National Institutes of Health Consensus Development Conference in 1994 [3]. A single course of corticosteroids such as dexamethasone or betamethasone has since been recommended for the management of imminent preterm birth, though the application of this practice varies throughout the world [4–6].

While the benefits of antenatal corticosteroid treatment to the neonates born preterm are well established, the potential harms to their mothers have drawn relatively little attention and remain mostly unclear. Despite the widespread use of antenatal corticosteroid treatment, emerging concerns have been raised about the potential harms, particularly after recent nationwide studies in the United States and Taiwan documented associations between short courses of oral corticosteroids and increased risks of sepsis, pneumonia, gastrointestinal bleeding, venous thromboembolism, heart failure, and fracture in the general population [7–9]. However, these studies did not focus on pregnant women, who are inherently at higher risk for several of these complications [10–12]. While one large population-based randomized controlled trial in six low- and middle-income countries showed a significant association of antenatal corticosteroid treatment with a 1.45 times increased risk of suspected maternal infections [13], few studies have addressed these concerns.

To fill this research gap, we employed a self-controlled case series design and used the nationwide population in Taiwan to quantify the association between antenatal corticosteroid treatment in pregnant women and the risks of severe adverse events, including sepsis, heart failure, and gastrointestinal bleeding.

## Methods

### Data sources

We used de-identified medical and pharmacy claims data derived from the entire National Health Insurance Research Database (NHIRD) and Birth Reporting Database (BRD) in Taiwan. Briefly, the data from NHIRD comprises the medical claims records of nearly 23 million enrollees, representing approximately 99% of the total population in Taiwan. Detailed descriptions of NHIRD have been published previously [8, 14, 15]. The BRD contains prenatal information such as birthweight, gestational age, type of delivery, and maternal age at delivery. The study protocol was approved by the Institutional Review Board of the National Health Research Institutes, Taiwan, and informed consent was waived because all data were encrypted.

### Study design and participants

**Fig. 1:**
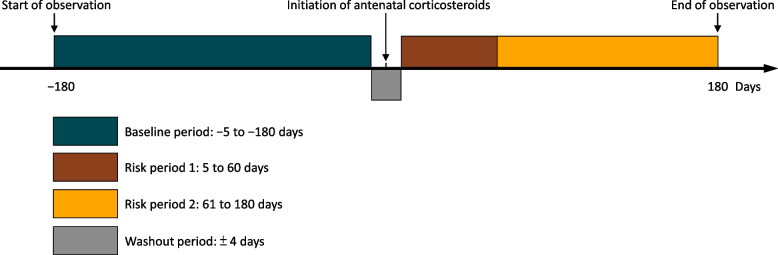
Illustration of study design and three observation periods (− 5 to − 180 days, 5 to 60 days, and 61 to 180 days) defined in this study

We applied a self-controlled case series (SCCS) design to determine the association between antenatal corticosteroid treatment and three severe adverse events in pregnant women: sepsis, heart failure, and gastrointestinal bleeding. One main strength of the SCCS design is that unmeasured time-invariant confounding effects are automatically eliminated in the subsequent analyses because the participants serve as their own controls [16]. We estimated the risk of each severe adverse event over three observation periods: one pre-treatment period (the baseline period defined as 5 ~ 180 days prior to initiation of antenatal corticosteroid treatment); and two post-treatment periods (days 5 ~ 60 and 61 ~ 180 after initiation of antenatal corticosteroid treatment). We compared the estimated risk during the pre-treatment period to the estimated risk at each of the post-treatment periods, separately, among participants exposed to antenatal corticosteroid treatment. We included a 4-day washout period during which observed severe adverse events were not considered since they might be caused by other factor(s). Figure 1 illustrates the study design and three observation periods defined in this study.

We included all women between 24 weeks 0/7 days to 36 weeks 6/7 days of gestation from 1 January 2009 to 31 December 2018, who were enrolled in the National Health Insurance Program at least 1 year prior to the study period. Inclusion criteria were participants who had no diagnosis of sepsis, heart failure, or gastrointestinal bleeding prior to the pre-treatment period, were with a singleton pregnancy, and had received a single course of antenatal steroid treatment for a pregnancy-related indication. A total of 52,119 pregnant women at 24 weeks 0/7 days to 36 weeks 6/7 days of gestation were included in this self-controlled case series study during the study period of 2009–2018.

### Antenatal corticosteroid treatment

The exposure of antenatal corticosteroids was identified from the prescription records included in the NHIRD. Antenatal corticosteroid treatment in Taiwan commonly consists of either betamethasone (two 12-mg doses administered intramuscularly 24 h apart) or dexamethasone (four 6-mg doses given intramuscularly 12 h apart), following the recommendations of the American College of Obstetricians and Gynecologists [4].

### Study outcomes

We investigated three severe adverse events potentially associated with antenatal corticosteroid treatment: sepsis, heart failure, and gastrointestinal bleeding. Episodes of migraine were treated as a negative control outcome. We identified the adverse events and negative control outcomes using ICD-9-CM codes for 2009–2015 and ICD-10-CM codes for 2016–2018 (Additional file 1: Table S1).

### Covariates

We treated oral corticosteroid use and three pregnancy complications (premature rupture of membranes, gestational diabetes, and gestational hypertension) as time-varying covariates in the subsequent analyses. Similarly, concomitant medication use for each severe adverse event was considered as time-varying covariates and adjusted correspondingly, for example, the use of nonsteroidal anti-inflammatory drugs (NSAIDs), aspirin, and systemic immunosuppressive agents for sepsis; the use of NSAIDs, antidiabetic drugs, antihypertensive drugs, antiplatelet drugs, bronchodilators, cardiac glycosides, hormone replacement therapy and nitrates for heart failure; and the use of NSAIDs, aspirin and proton pump inhibitors for gastrointestinal bleeding.

### Statistical analysis

Baseline demographic and clinical characteristics are provided in Table 1. We defined participants with antenatal corticosteroid treatment, and participants without any antenatal corticosteroid treatment during the entire study period. We estimated incidence rates per 1000 person-years of the three severe adverse events for participants with and without antenatal corticosteroid treatment (Table 2). Conditional Poisson regression was used to estimate incidence rate ratios (IRRs) to compare the incidence rates of each adverse event and the negative control outcome in each post-treatment period to those for the baseline period. We adjusted for the time-varying covariates described above in the regression models. To investigate the robustness of the observed results, we conducted sensitivity analyses to evaluate the potential influence of various durations (a 7-day washout period; and the baseline period of 5 ~ 120 days prior to initiation of antenatal corticosteroid treatment versus two post-treatment periods of 5 ~ 30 and 31 ~ 120 days after initiation of antenatal corticosteroid treatment), gestation at 24 weeks 0/7 days to 33 weeks 6/7 days, independence of study events, exposure to two or more courses of antenatal corticosteroid treatment; and no history of preeclampsia, premature rupture of the membranes and peripartum cardiomyopathy, respectively. *E*-value measurement was employed to evaluate potential unmeasured confounding [17]. Subgroup analysis was carried out to examine potential differential effects of preterm (< 37 weeks of gestation) versus term (> 37 weeks of gestation) delivery, and the two most common kinds of corticosteroids used during the antenatal period: betamethasone and dexamethasone. All analyses were performed using SAS version 9.4 (SAS Institute, Cary, NC, USA).

**Table 1:** Baseline demographic and clinical characteristics of study participants with antenatal corticosteroid treatment

Characteristic	*N* = 52,119
**Age, mean (SD), year**	31.91 (5.24)
**Gestational weeks at delivery, mean (SD), week**	34.93 (3.33)
**Parity**	
1	21,322 (40.91)
2	24,489 (46.99)
3	5309 (10.19)
≥ 4	999 (1.91)
**Delivery mode, *n* (%)**	
Vaginal	26,110 (50.10)
Cesarean	26,009 (49.90)
**Preterm delivery, *n* (%)**	
No	19,728 (37.85)
Yes	32,391 (62.15)
**Premature rupture of membranes, *n* (%)**	
No	35,199 (67.54)
Yes	16,920 (32.46)
**Gestational diabetes, *n* (%)**	
No	40,784 (78.25)
Yes	11,335 (21.75)
**Gestational hypertension, *n* (%)**	
No	44,411 (85.21)
Yes	7708 (14.79)

*Abbreviation*: *SD* standard deviation

**Table 2:** Incidence rates of sepsis, heart failure, and gastrointestinal bleeding in participants with and without antenatal corticosteroid treatment

	**With antenatal corticosteroid treatment**			**Without antenatal corticosteroid treatment**			**Rate difference/1000 person-years (95% CI)**
Event	No. of cases	No. of person-years	Incidence rate per 1000 person-years (95% CI)	No. of cases	No. of person-years	Incidence rate per 1000 person-years (95% CI)	
Sepsis	418	551,556	0.76 (0.69–0.83)	7773	15,831,237	0.49 (0.48–0.50)	0.27 (0.19–0.34)
Heart failure	173	552,754	0.31 (0.27–0.36)	1564	15,865,236	0.10 (0.09–0.10)	0.21 (0.17–0.26)
GI bleeding	5854	506,126	11.57 (11.27–11.87)	137,962	14,820,062	9.31 (9.26–9.36)	2.26 (1.96–2.56)

*Abbreviation*: *CI* confidence interval, *GI* gastrointestinal

## Results

### Demographic and clinical characteristics of study participants at baseline

A total of 52,119 eligible participants during the study period from 2009 to 2018 were included. Table 1 reports the baseline demographic and clinical characteristics of the included study participants. The mean age at baseline was 31.9 ± 5.2 years; mean gestational week at delivery was 34.9 ± 3.3 weeks; 32.5% had premature rupture of membranes; 21.8% had gestational diabetes; and 14.8% had gestational hypertension (Table 1). In addition, the characteristics of participants with and without antenatal corticosteroid treatment were presented (Additional file 1: Table S2). Compared to participants without antenatal corticosteroid treatment, participants with antenatal corticosteroid treatment had shorter gestational weeks at delivery and higher proportions of cesarean delivery, preterm delivery, premature rupture of membranes, gestational diabetes, and gestational hypertension.

### Incidence rates of the three adverse events

The incidence rates per 1000 person-years of the three severe adverse events (sepsis, heart failure, and gastrointestinal bleeding) for participants with and without antenatal corticosteroid treatment are provided in Table 2. The incidence rates among participants who received antenatal corticosteroid treatment were significantly greater than among those who did not receive antenatal corticosteroid treatment. The incidence rate differences per 1000 person-years between the two groups were 0.27 [95% CI, 0.19–0.34] for sepsis, 0.21 [95% CI, 0.17–0.26] for heart failure, and 2.26 [95% CI, 1.96–2.56] for gastrointestinal bleeding (Table 2).

### Risks of the three severe adverse events

**Fig. 2:**
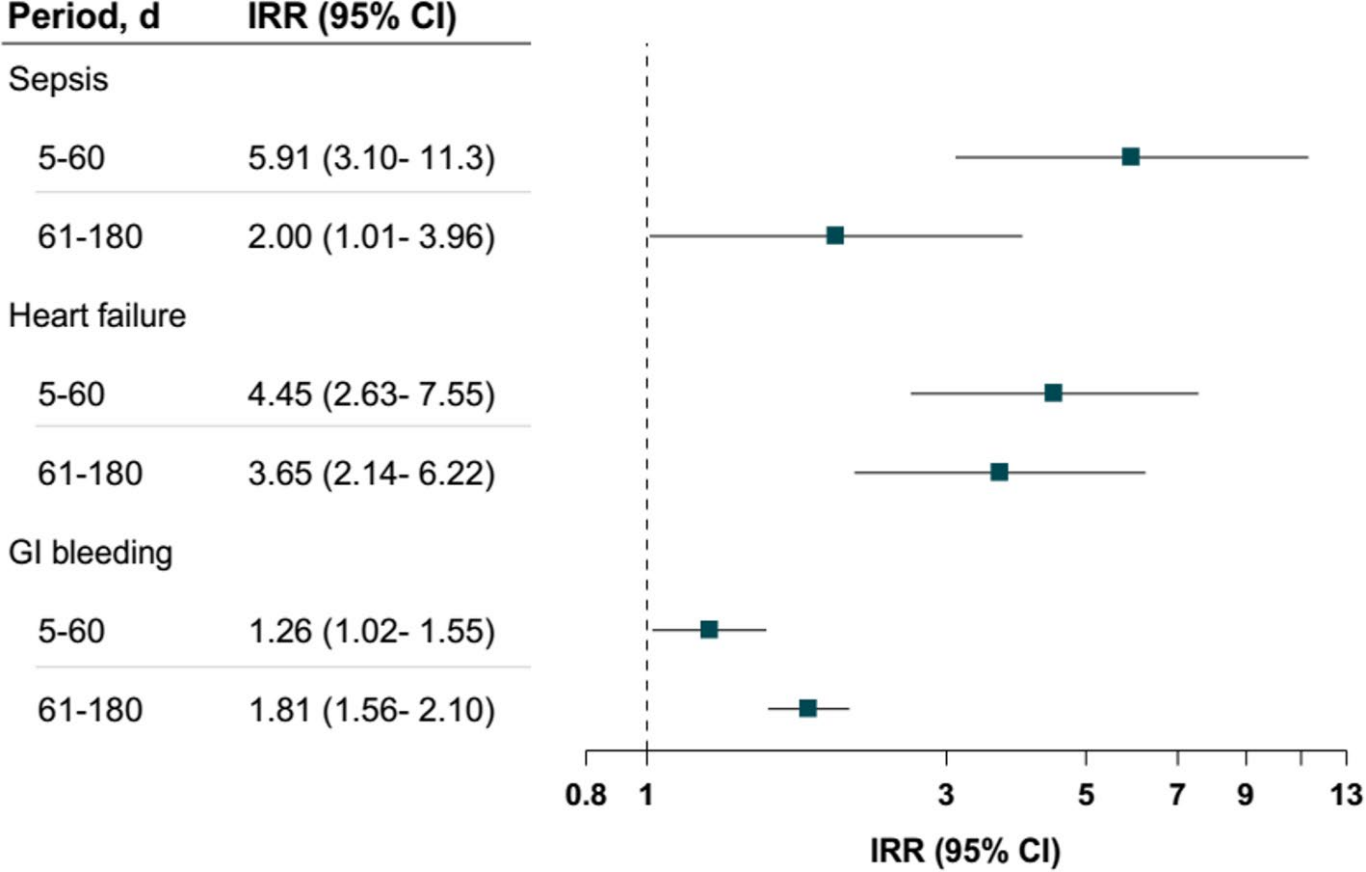
Association between antenatal corticosteroid treatment and three severe adverse events (sepsis, heart failure, and GI bleeding) during 2 post-treatment periods (5–61 and 61–180 days). IRR, incidence risk ratio; CI, confidence interval; GI, gastrointestinal

Figure 2 indicates that the IRRs for sepsis and heart failure among participants with antenatal corticosteroid treatment during the first post-treatment period (5 ~ 60 days after administering antenatal corticosteroids) were significantly higher than during the baseline period (IRR: 5.91; 95% CI: 3.10–11.30 for sepsis; IRR: 4.45; 95% CI: 2.63–7.55 for heart failure; IRR: 1.26; 95% CI: 1.02–1.55 for gastrointestinal bleeding). Likewise, significant IRRs for the three adverse events during the second post-treatment period (61 ~ 180 days after treating antenatal corticosteroids) were also observed (IRR: 2.00; 95% CI: 1.01–3.96 for sepsis and 3.65; 95% CI: 2.14–6.22 for heart failure; IRR: 1.81; 95% CI: 1.56–2.10 for gastrointestinal bleeding). The results showed no association between antenatal corticosteroid treatment and the risk of migraine, the negative control outcome (Additional file 1: Table S3).

### Sensitivity analyses

We performed a series of sensitivity analyses to investigate the impact of the different durations of the washout and observation periods, different lengths of gestation (24 weeks 0/7 days to 33 weeks 6/7 days), independence of study events, exposure to two or more courses of antenatal corticosteroid treatment; and no history of preeclampsia, premature rupture of the membranes and peripartum cardiomyopathy, respectively. The results indicated that participants with two or more courses of antenatal corticosteroid treatment tended to have higher risks of sepsis and heart failure than those with a single course, but the differences did not reach statistical significance. The observed results from the other sensitivity analyses were comparable to the findings from the main analysis (Table 3 and Additional file 1: Fig. S1). The estimated *E*-values for sepsis, heart failure, and gastrointestinal bleeding ranged from 1.83 to 11.30 in the first post-treatment period, and from 3.02 to 6.76 in the second post-treatment period, showing no considerable unmeasured confounding (Additional file 1: Table S4).

**Table 3:** Sensitivity analyses to investigate adverse events associated with antenatal corticosteroid treatment

Adverse event	Events, *n*	Incidence rate ratio (95% CI)			
		**Crude**		**Adjusted**^a^	
		**5–60 days**	**61–180 days**	**5–60 days**	**61–180 days**
**Pregnant women at 24 weeks 0/7 days to 33 weeks 6/7 days of gestation**					
Sepsis	46	**5.24** (2.56–10.7)	1.71 (0.79–3.70)	**4.48** (2.16–9.28)	1.47 (0.67–3.21)
Heart failure	89	**6.11** (3.46–10.8)	**2.93** (1.67–5.17)	**3.98** (2.18–7.30)	**3.38** (1.85–6.18)
GI bleeding	678	1.24 (0.98–1.58)	**2.18** (1.85–2.58)	1.13 (0.89–1.44)	**1.68** (1.42–1.99)
**Adverse events are independent**^b^					
Sepsis	57	**9.08** (4.25–19.4)	**2.77** (1.23–6.21)	**8.61** (4.01–18.5)	**2.62** (1.16–5.92)
Heart failure	97	**11.7** (6.02–22.8)	1.33 (0.57–3.14)	**11.2** (5.33–23.4)	2.31 (0.92–5.78)
GI bleeding	683	**1.74** (1.24–2.45)	**2.33** (1.79–3.02)	**1.60** (1.13–2.26)	**1.80** (1.38–2.36)
**Two or more courses of antenatal corticosteroid treatment**					
Sepsis	11	**12.6 (1.41–112)**	**8.8 (1.06–73.1)**	**12.8 (1.43–114)**	**8.90 (1.07–73.9)**
Heart failure	22	**5.50 (1.61–18.8)**	**4.03 (1.28–12.7)**	**4.95 (1.44–17.1)**	**3.74 (1.19–11.8)**
GI bleeding	93	0.71 (0.36–1.42)	1.30 (0.85–2.00)	0.71 (0.36–1.41)	1.30 (0.84–2.01)
**Exclusion of pregnancy conditions**^c^					
Sepsis	37	**17.3 (5.96–50.2)**	**4.03 (1.28–12.7)**	**16.8 (5.58–50.5)**	**3.84 (1.18–12.5)**
Heart failure	75	**4.47 (2.48–8.03)**	**2.24 (1.26–3.99)**	**3.95 (2.12–7.33)**	**3.33 (1.78–6.22)**
GI bleeding	760	**1.37 (1.08–1.72)**	**2.60 (2.22–3.05)**	**1.29 (1.02–1.63)**	**1.91 (1.62–2.26)**

*Abbreviation*: *CI* confidence interval, *GI* gastrointestinal

^a^Sepsis was adjusted for premature rupture of membranes, gestational diabetes, gestational hypertension, oral corticosteroid (OCS), non*-*steroidal anti*-*inflammatory drugs (NSAIDs), aspirin, systemic immunosuppressive agents, and acute conditions; heart failure was adjusted for premature rupture of membranes, gestational diabetes, gestational hypertension, OCS, NSAIDs, hormone replacement therapy, bronchodilators, antidiabetic drugs, cardiac glycosides, antihypertensive drugs, nitrates, antiplatelet drugs, and acute conditions; and GI bleeding was adjusted for premature rupture of membranes, gestational diabetes, gestational hypertension, OCS, NSAIDs, aspirin and proton pump inhibitors, and acute conditions

^b^To ensure consecutive events are independent, only the first event in each observation period was selected

^c^Excluded pregnancy conditions were preeclampsia, premature rupture of the membranes, and peripartum cardiomyopathy

### Subgroup analyses

We performed subgroup analyses stratified by gestational age at delivery and the two different kinds of corticosteroids. Comparable risks were found between the group who delivered at term versus at preterm (Additional file 1: Table S5). The results from the subgroup analysis comparing antenatal betamethasone with antenatal dexamethasone treatment are shown (Additional file 1: Fig. S2). Compared to the baseline period, similar risks of sepsis, heart failure, and gastrointestinal bleeding for each of the two post-treatment periods were found between the group receiving antenatal betamethasone and those receiving antennal dexamethasone.

## Discussion

In this nationwide population-based SCCS study in which 52,119 pregnant women were exposed to antenatal corticosteroid treatment, we found that a single course of antenatal corticosteroids was significantly associated with a 5.9-, 4.5-, and 1.3-fold increased risk of sepsis, heart failure, and gastrointestinal bleeding, respectively. The highest risk for sepsis and heart failure occurred within the first 2 months after receipt of antenatal corticosteroids, while the highest risk for gastrointestinal bleeding occurred in the third to sixth months after treatment. The results were robust across several sensitivity analyses including different durations of washout and observation periods, different lengths of gestation (24 weeks 0/7 days to 33 weeks 6/7 days), independence of the study events, and exposure to two or more courses of antenatal corticosteroid treatment. Subgroup analyses showed comparable risks of severe adverse events in pregnant women who delivered at preterm or at term and who received treatment with antenatal betamethasone or dexamethasone. To our knowledge, this is the first nationwide SCCS study to demonstrate the association of antenatal corticosteroid treatment with risks of severe adverse events in pregnant women.

The findings suggest several implications. First, while the benefits of antenatal corticosteroid treatment for fetal maturation are well recognized, the lurking risks of severe adverse events in pregnant women remain unobserved. This study provides real-world evidence that antenatal corticosteroid treatment is not harmless but poses potentially serious health risks to pregnant women. The findings not only reveal the potential risk of sepsis related to antenatal corticosteroid treatment, which is in line with previous studies [13, 18, 19], but also highlight a novel positive association of antenatal corticosteroid treatment with heart failure and gastrointestinal bleeding. Second, our findings suggest that the increased risks of sepsis, heart failure, and gastrointestinal bleeding persist up to 180 days after receipt of antenatal corticosteroid treatment—well into the postpartum period for all the women studied. It is important for clinicians to be aware of the potential harms of antenatal corticosteroid treatment to pregnant women, especially during the first several months of corticosteroid administration. Third, since the fetal benefits of antenatal corticosteroids make their continued use likely, our findings underscore the need for physicians and policymakers to incorporate maternal health considerations, including recommendations for adverse event monitoring, into future guidelines for antenatal corticosteroid treatment.

We evaluated the relative risks for three observation periods, one pre-treatment and two post-treatment periods, which approximately correspond to the pregnancy, peripartum, and postpartum periods, respectively. Our study found that the highest risk for sepsis and heart failure occurred within the first post-treatment period (the peripartum period) after antenatal corticosteroid treatment. While this may be due to treatment effect alone, previous studies suggest an increased risk of sepsis and heart failure during the peripartum period relative to the remainder of pregnancy, even in women not exposed to corticosteroids [11, 20]. Our *E*-values for sepsis and heart failure suggest it is less likely that unmeasured confounding fully explains our results, and we note that the observed risks also persisted up to 180 days after receipt of antenatal corticosteroids, well into the postpartum period. Still, the observed increase in risks of sepsis and heart failure, especially during the first 2 months after antenatal corticosteroid exposure, should be interpreted with caution.

In contrast to sepsis and heart failure, the highest risk for gastrointestinal bleeding occurred at 3 to 6 months after antenatal corticosteroid exposure. Initially, it seems counterintuitive that the risk of an adverse event would be higher 3 to 6 months after an exposure than it would immediately post-exposure. However, pregnancy is a naturally hypercoagulable state due to elevated platelet aggregation, which could prevent hemorrhage during pregnancy [21, 22]. As such, the observed risk for gastrointestinal bleeding related to antenatal corticosteroid treatment may subsequently arise as the hypercoagulable state of pregnancy progressively resolves over 4 to 6 weeks after childbirth.

Dexamethasone and betamethasone are the most widely used corticosteroids prescribed for use among pregnant women at risk of delivering preterm babies [23]. Comparative effectiveness and safety between these two corticosteroids are unclear. Currently, there is no conclusive evidence to support that one is superior to the other—either for a neonate or a pregnant woman. A multicenter randomized controlled trial in Australia and New Zealand showed similar effects of betamethasone and dexamethasone treatment on infant health outcomes [23]. A retrospective cohort study suggested that antenatal betamethasone treatment was associated with lower rates of respiratory distress syndrome among neonates than antenatal dexamethasone [24]. However, another network meta-analysis indicated that dexamethasone was associated with a marginally reduced risk of chorioamnionitis among pregnant women, compared to betamethasone [25]. Our subgroup analysis found comparable maternal risks of the three adverse events related to the use of dexamethasone and betamethasone, respectively, providing initial data to support further investigations.

This study has several strengths. We used the entire national medical claims database in Taiwan; thus, the large sample size provides adequate statistical power to evaluate the effect of antenatal corticosteroid treatment on three severe adverse events in pregnant women. Because of the universal health care program deployed in Taiwan, we are able to capture data for nearly every pregnant woman. Hence, the extent of potential recall or selection bias is minimized. The use of the SCCS design in this study automatically eliminates time-invariant confounding effects. Nevertheless, several limitations should be noted. First, as with all observational studies, the possibility of unmeasured confounding effects cannot be ruled out. Based on the *E*-values for the three severe adverse events, it is less likely that unmeasured confounding can explain the observed risk for those three adverse events. Secondly, we were not able to adjust for maternal pre-pregnancy body mass index in the models because it is not available in the NHIRD. Even though the *E*-values indicated a limited extent of unmeasured confounding, and we adjusted for NSAIDs use in the models as well as excluded serious maternal complications, the influences of unmeasured confounding and confounding by indication cannot be totally ruled out. Hence, the observed results should be interpreted with caution. Third, we only examined three severe adverse events linked to antenatal corticosteroid treatment. It would be interesting to evaluate whether the effects of antenatal corticosteroid treatment can be generalized to additional health outcomes in women. Fourth, when stratified by preterm and term deliveries, most associations did not reach statistical significance in the term group, though they were in the same direction in the preterm and term groups. There may have been limited statistical power due to a small number of adverse events in the term group. Fifth, this study only investigated the effect of antenatal corticosteroid treatment in a population of Asian women. Further investigation is needed to validate the observed risks of three adverse events across different populations.

## Conclusion

This nationwide SCCS study provides real-world evidence that a single course of antenatal corticosteroids among pregnant women is significantly associated with a 1.3- to 5.9-fold increased risk of sepsis, heart failure, and gastrointestinal bleeding. Our findings suggest that providers should carefully weigh the potential risks and benefits of antenatal corticosteroid treatment when prescribed for pregnant women. Additional studies, including randomized clinical trials or prospective cohort studies, are warranted to better understand avoidable harms from the use of antenatal corticosteroid treatment.

### Supplementary Information

**Additional file 1: Figure S1.** Association between antenatal corticosteroid treatment and three severe adverse events (sepsis, heart failure and GI bleeding) based on various durations of washout and post-treatment periods. IRR = incidence risk ratio; CI = confidence interval; GI = gastrointestinal. **Figure S2.** Association between antenatal corticosteroid treatment and three severe adverse events (sepsis, heart failure and GI bleeding) stratified by treatment with betamethasone and dexamethasone, separately. IRR = incidence risk ratio; CI = confidence interval; GI = gastrointestinal. **Table S1.** ICD-9-CM and ICD-10-CM codes of the three adverse events and negative control outcome. **Table S2.** Baseline characteristics of study participants with and without antenatal corticosteroid treatment. **Table S3.** Incidence rate ratios for migraine (negative control outcome) associated with antenatal corticosteroid treatment. **Table S4.** Incidence rate ratios and E-values for three adverse events associated with antenatal corticosteroid treatment. **Table S5.** Association between antenatal corticosteroid treatment and three severe adverse events (sepsis, heart failure and GI bleeding) stratified by preterm versus full-term delivery.

## Data Availability

De-identified participant data from the NHIRD of Taiwan are managed by the Taiwan Ministry of Health and Welfare. No additional data are available.
