# Supplementary material for: Association between antenatal corticosteroid treatment and severe adverse events in pregnant women
Source: BMC Med. 2023 Oct 31;21:413. doi: 10.1186/s12916-023-03125-w (PMC10617183; doi:10.1186/s12916-023-03125-w)
Supplement: Supplementary file 1 — Additional file 1: Figure S1. Association between antenatal corticosteroid treatment and three severe adverse events (sepsis, heart failure and GI bleeding) based on various durations of washout and post-treatment periods. IRR = incidence risk ratio; CI = confidence interval; GI = gastrointestinal. Figure S2. Association between antenatal corticosteroid treatment and three severe adverse events (sepsis, heart failure and GI bleeding) stratified by treatment with betamethasone and dexamethasone, separately. IRR = incidence risk ratio; CI = confidence interval; GI = gastrointestinal. Table S1. ICD-9-CM and ICD-10-CM codes of the three adverse events and negative control outcome. Table S2. Baseline characteristics of study participants with and without antenatal corticosteroid treatment. Table S3. Incidence rate ratios for migraine (negative control outcome) associated with antenatal corticosteroid treatment. Table S4. Incidence rate ratios and E-values for three adverse events associated with antenatal corticosteroid treatment. Table S5. Association between antenatal corticosteroid treatment and three severe adverse events (sepsis, heart failure and GI bleeding) stratified by preterm versus full-term delivery. [file 12916_2023_3125_MOESM1_ESM.docx]

**Table S1.** ICD-9-CM and ICD-10-CM codes of the three adverse events and negative control outcome.

| **Diagnosis** | **ICD-9-CM Code** | **ICD-10-CM Code** |
| --- | --- | --- |
| **Adverse events** | | |
| **Sepsis** | 038.xx, 785.52, 998.02,  995.90- 995.94 | A40.xxxx, A41.xxxx, R65.10xx, R65.11xx, R65.20xx, R65.21xx, T81.12XA, T81.12XD, T81.12XS |
| **Heart failure** | 428.xx | I50.1x, I50.9x, I50.20- I50.23,  I50.30- I50.33, I50.40-I50.43 |
| **GI bleeding** | 530.7x, 531.xx, 531.2x, 531.4x, 531.6x, 532.xx, 532.2x, 532.4x, 532.6x, 533.xx, 533.2x, 533.4x, 533.6x, 534.xx, 534.2x, 534.4x, 534.6x, 569.3x, 535.01, 535.11, 535.21, 535.31, 535.41, 535.51, 535.61, 535.71, 537.83, 537.84, 562.02, 562.03, 562.12, 562.13, 569.85, 578.xx | K22.6xxx, K25.xxxx-K28.xxxx, K29.01xx, K29.21xx, K29.31xx, K29.41xx, K29.51xx, K29.61xx, K29.71xx, K29.81xx, K29.91xx, K31.811x, K31.82xx, K52.81xx, K55.21xx, K56.60xx, K57.01xx, K57.11xx, K57.13xx, K57.21xx, K57.31xx, K57.33xx, K57.81xx, K57.91xx, K57.93xx, K62.5xxx, K92.0xxx-K92.2xxx |
| **Negative control outcome** | | |
| **Migraine** | 346.xx | G43.xxxx |

Note:

Abbreviation: **GI**: gastrointestinal; **ICD-9-CM**: International Classification of Diseases, Ninth Revision, Clinical Modification; **ICD-10-CM**: International Classification of Diseases, Tenth Revision, Clinical Modification.

**Table S2.** Baseline characteristics of study participants with and without antenatal corticosteroid treatment.

| **Characteristic** | **With antenatal corticosteroid treatment**  ***n* = 52,119** | **Without antenatal corticosteroid treatment**  *n* = **2,105,202** |
| --- | --- | --- |
| **Age, mean (SD), year** | 31.91 (5.24) | 31.23 (4.88) |
| **Gestational weeks at delivery, mean (SD), week** | 34.93 (3.33) | 38.41 (1.41) |
| **Parity** |  |  |
| 1 | 21,322 (40.91) | 807,625 (38.36) |
| 2 | 24,489 (46.99) | 1,060,272 (50.36) |
| 3 | 5,309 (10.19) | 205,694 (9.77) |
| ≥ 4 | 999 (1.91) | 31,611 (1.50) |
| **Delivery mode, n (%)** |  |  |
| Vaginal | 26,110 (50.10) | 1,393,607 (66.20) |
| Cesarean | 26,009 (49.90) | 711,595 (33.80) |
| **Preterm delivery, *n* (%)** |  |  |
| No | 19,728 (37.85) | 1,986,113 (94.34) |
| Yes | 32,391 (62.15) | 119,089 (5.66) |
| **Premature rupture of membranes, n (%)** |  |  |
| No | 35,199 (67.54) | 1,911,219 (90.79) |
| Yes | 16,920 (32.46) | 193,983 (9.21) |
| **Gestational diabetes, n (%)** |  |  |
| No | 40,784 (78.25) | 1,757,178 (83.47) |
| Yes | 11,335 (21.75) | 348,024 (16.53) |
| **Gestational hypertension, n (%)** |  |  |
| No | 44,411 (85.21) | 2,011,468 (95.55) |
| Yes | 7,708 (14.79) | 93,734 (4.45) |

Note:

Abbreviation: **SD**: standard deviation.

**Table S3.** Incidence rate ratios for migraine (n**egative control outcome)** associated with antenatal corticosteroid treatment.

| **Adverse Event** | **Events, *n*** | **Incidence Rate Ratio (95% CI)**^a^ | |
| --- | --- | --- | --- |
|  |  | **5-60 Days** | **61-180 Days** |
| **Migraine** | 249 | 0.67 (0.45-1) | 1.02 (0.78-1.33) |

Note:

Abbreviation: **CI**: confidence interval.

^a^Model was adjusted for premature rupture of membranes, gestational diabetes, gestational hypertension and oral corticosteroid use.

**Table S4.** Incidence rate ratios and E-values for three adverse events associated with antenatal corticosteroid treatment.

| **Adverse Event** | **Events, *n*** | **5-60 Days** | | **61-180 Days** | |
| --- | --- | --- | --- | --- | --- |
|  |  | **Incidence Rate Ratio (95% CI)** | **E-value (CI**^a^**)** | **Incidence Rate Ratio (95% CI)** | **E-value (CI**^a^**)** |
| **Sepsis**^b^ | 64 | 5.91 (3.10- 11.3) | 11.30 (5.65) | 2.00 (1.01- 3.96) | 3.41 (1.11) |
| **Heart failure**^b^ | 111 | 4.45 (2.63- 7.55) | 8.37 (4.70) | 3.65 (2.14- 6.22) | 6.76 (3.70) |
| **GI bleeding**^b^ | 930 | 1.26 (1.02- 1.55) | 1.83 (1.16) | 1.81 (1.56- 2.10) | 3.02 (2.49) |

Note:

Abbreviation: **CI**: confidence interval; **GI**: gastrointestinal.

^a^E-values for lower limit of the CI when RR>1; E-values for upper limit of the CI when RR<1.

^b^**Sepsis** was adjusted for premature rupture of membranes, gestational diabetes, gestational hypertension, oral corticosteroid (OCS), non*-*steroidal anti*-*inflammatory drugs (NSAIDs), aspirin, systemic immunosuppressive agents and acute conditions; **heart failure** was adjusted for premature rupture of membranes, gestational diabetes, gestational hypertension, OCS, NSAIDs, hormone replacement therapy, bronchodilators, antidiabetic drugs, cardiac glycosides, antihypertensive drugs, nitrates, antiplatelet drugs and acute conditions; and **GI bleeding** was adjusted for premature rupture of membranes, gestational diabetes, gestational hypertension, OCS, NSAIDs, aspirin and proton pump inhibitors and acute conditions.

**Table S5.** Association between antenatal corticosteroid treatment and three severe adverse events (sepsis, heart failure and GI bleeding) stratified by preterm versus full-term delivery.

| **Adverse Event** | **Preterm**  (*n*=32,391) | | | **Term**  (*n*=19,728) | | |
| --- | --- | --- | --- | --- | --- | --- |
|  | **Incidence Rate Ratio (95% CI)** | | | | | |
|  | **Events, *n*** | **Crude** | **Adjusted**^a^ | **Events, *n*** | **Crude** | **Adjusted**^a^ |
|  |  | **5-60 Days** | **61-180 Days** |  | **5-60 Days** | **61-180 Days** |
| **Sepsis** | 29 | **6.66 (3.24-13.7)** | 2.04 (0.94- 4.41) | 8 | 1.55 (0.30- 8.04) | 0.97 (0.21- 4.54) |
| **Heart failure** | 62 | **4.89 (2.73- 8.75)** | **4.13 (2.30- 7.45)** | 13 | 2.45 (0.65- 9.22) | 1.53 (0.40- 5.82) |
| **GI bleeding** | 449 | 1.25 (0.96- 1.62) | **1.89 (1.56- 2.29)** | 311 | 1.33 (0.94- 1.87) | **1.73 (1.35- 2.21)** |

Note:

Abbreviation: **CI**: confidence interval; **GI**: gastrointestinal.

^a^**Sepsis** was adjusted for premature rupture of membranes, gestational diabetes, gestational hypertension, oral corticosteroid (OCS), non*-*steroidal anti*-*inflammatory drugs (NSAIDs), aspirin, systemic immunosuppressive agents and acute conditions; **heart failure** was adjusted for premature rupture of membranes, gestational diabetes, gestational hypertension, OCS, NSAIDs, hormone replacement therapy, bronchodilators, antidiabetic drugs, cardiac glycosides, antihypertensive drugs, nitrates, antiplatelet drugs and acute conditions; and **GI bleeding** was adjusted for premature rupture of membranes, gestational diabetes, gestational hypertension, OCS, NSAIDs, aspirin and proton pump inhibitors and acute conditions.

**
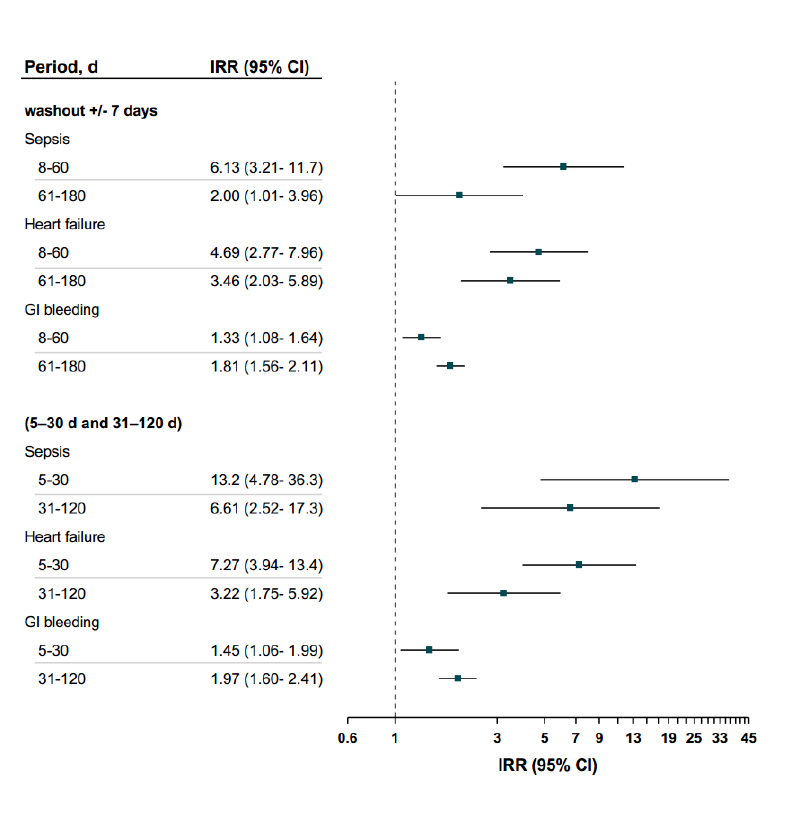
**

**Figure S1.** Association between antenatal corticosteroid treatment and three severe adverse events (sepsis, heart failure and GI bleeding) based on various durations of washout and post-treatment periods. **IRR** = incidence risk ratio; **CI** = confidence interval; **GI** = gastrointestinal.


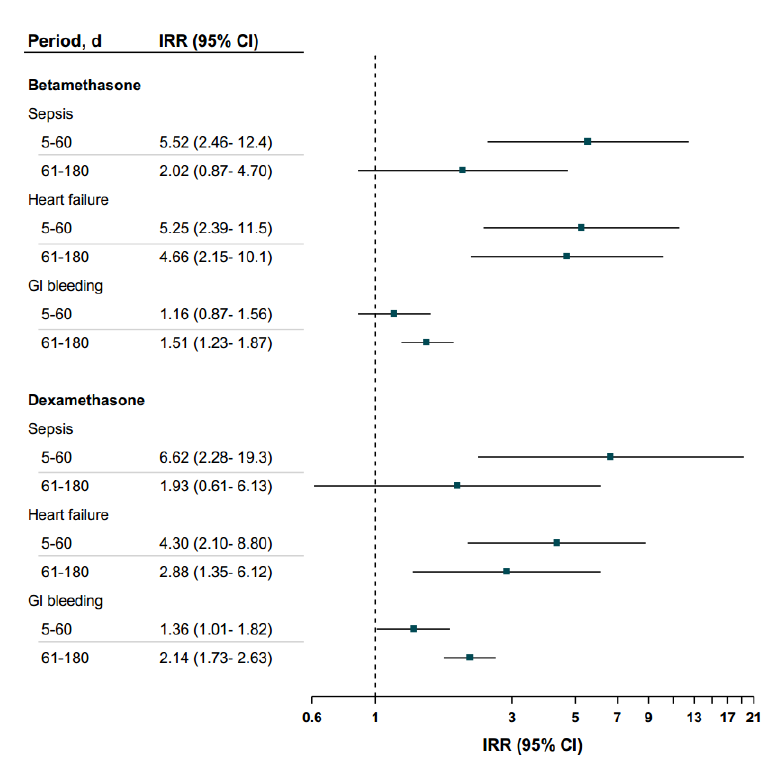


**Figure S2.** Association between antenatal corticosteroid treatment and three severe adverse events (sepsis, heart failure and GI bleeding) stratified by treatment with betamethasone and dexamethasone, separately. **IRR** = incidence risk ratio; **CI** = confidence interval; **GI** = gastrointestinal.
